# Synthesis, characterization, X-ray structure, computational studies, and bioassay of novel compounds combining thiophene and benzimidazole or 1,2,4-triazole moieties

**DOI:** 10.1186/s13065-017-0280-6

**Published:** 2017-06-09

**Authors:** Yahia N. Mabkhot, Salim S. Al-Showiman, Saied M. Soliman, Hazem A. Ghabbour, Murad A. AlDamen, Mohammad S. Mubarak

**Affiliations:** 1 Department of Chemistry, College of Science, King Saud University, P.O. Box 2455, Riyadh, 11451 Saudi Arabia; 2 Department of Chemistry, College of Science & Arts, King Abdulaziz University, P.O. Box 344, Rabigh, 21911 Saudi Arabia; 3 Department of Chemistry, Faculty of Science, Alexandria University, P.O. Box 426, Ibrahimia, Alexandria, 21321 Egypt; 4 Department of Pharmaceutical Chemistry, College of Pharmacy, King Saud University, P.O. Box 2457, Riyadh, 11451 Saudi Arabia; 5 Department of Chemistry, The University of Jordan, Amman, 11942 Jordan

**Keywords:** Thiophene-containing compounds, X-ray diffraction, DFT, Antibacterial and antifungal activity, Molecular structure

## Abstract

**Background:**

Due to their interesting and versatile biological activity, thiophene-containing compounds have attracted the attention of both chemists and medicinal chemists. Some of these compounds have anticancer, antibacterial, antiviral, and antioxidant activity. In addition, the thiophene nucleus has been used in the synthesis of a variety of heterocyclic compounds.

**Results:**

In the present work, two novel thiophene-containing compounds, 4-phenyl-2-phenylamino-5-(1*H*-1,3-a,8-triaza-cyclopenta[α]inden-2-yl)-thiophene-3-carboxylic acid ethyl ester (**3**) and 5-(1*H*-Imidazo[1,2-b] [1,2,4] triazol-5-yl)-4-phenyl-2-phenylamino-thiophene-3-carboxylic acid ethyl ester (**4**), have been synthesized by reaction of 5-(2-bromo-acetyl)-4-phenyl-2-phenylaminothiophene-3-carboxylic acid ethyl ester (**2**) with 2-aminobenzimidazole and 3-amino-1*H*-1,2,4-triazole in the presence of triethylamine, respectively. Compound **2**, on the other hand, was prepared by bromination of 5-acetyl-4-phenyl-2-phenylaminothiophene-3-carboxylic acid ester (**1**). Structures of the newly prepared compounds were confirmed by different spectroscopic methods such as ^1^H-NMR, ^13^C-NMR, and mass spectrometry, as well as by elemental analysis. Furthermore, bromination of compound **1** led to the formation of two constitutional isomers (**2a** and **2b**) that were obtained in an 80:20 ratio. Molecular structures of **2b** were confirmed with the aid of X-ray crystallography. Compound **2** was crystallized in the triclinic, *P*-1, *a* = 8.8152 (8) Å, *b* = 10.0958 (9) Å, *c* = 12.6892 (10) Å, α = 68.549 (5)°, *β* = 81.667 (5)°, γ = 68.229 (5)°*, V* = 976.04 (15) Å^3^, *Z* = 2, and was found in two isomeric forms regarding the position of the bromine atom. The antibacterial and antifungal activities of the prepared compounds were evaluated.

**Conclusions:**

Three new thiophene derivatives were synthesized in good yield. Antimicrobial screening revealed that compound **3** was a promising candidate as a potential antibacterial and antifungal agent; it exhibits remarkable activity against the studied bacterial strains, especially the gram negative bacteria *E. coli* in addition to some fungi. More work is needed to evaluate its safety and efficacy.

## Background

**Fig. 1:**
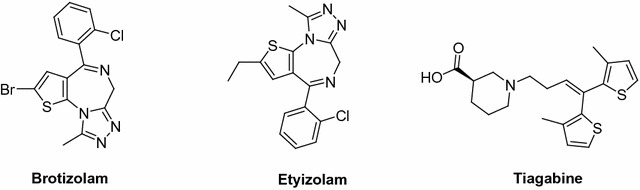
Structures of some bioactive compounds containing thiophene moiety

For the past several years, thiophene-containing compounds have gained popularity in the field of organic and medicinal chemistry, and have attracted tremendous interest among organic and medicinal chemists owing to their remarkable and wide range of biological activities, such as antidepressant [1], analgesic [2], anti-inflammatory [3], anticonvulsant [4–7], and other antimicrobial properties [8]. In addition, the thiophene moiety is central in the structure of different antiepileptic drugs (AEDs) such as brotizolam [9], etizolam [10], and tiagabine [11], structures of which are shown in Fig. 1. Very recently, we have reported on the synthesis, X-ray structure, and bioactivity of new thiophene-containing compounds [11, 12]. We have described the synthesis, X-ray structure, and calculations pertaining to the new compound, (*2E,2′E*)-1,1′-(3,4-diphenylthieno [2,3-*b*] thiophene-2,5-diyl) bis (3-(dimethylamino)prop-2-en-1-one) [11]. In addition, we have prepared and characterized a number of novel thieno [2,3-*b*] thiophene derivatives and have evaluated their bioactivity against fungi and gram-negative bacteria [12].

**Scheme 1:**
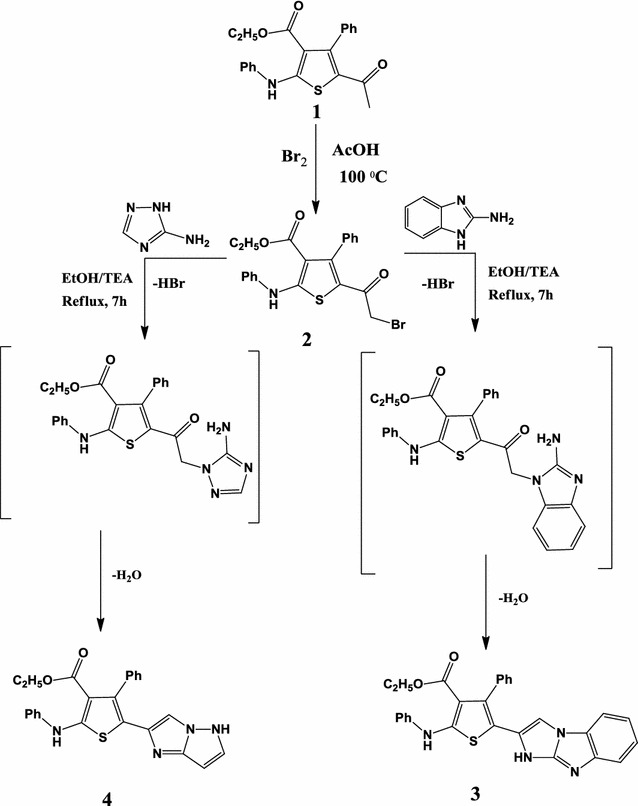
Synthesis of compounds **2**, **3**, and **4**

As part of our ongoing research in the synthesis of new heterocyclic compounds containing a thiophene core (Scheme 1), we describe herein the synthesis, characterization, and X-ray structure determination of novel thiophene-containing compounds. In addition, we found that compound **2** was formed in two isomeric forms; **2a** where the bromine atom is on the side chain, and **2b**, where the bromine is attached to the benzene ring. We performed energy analysis and explored other thermodynamic parameters on the two structural isomers **2a** and **2b** to account for the stability of one over the other. Furthermore, we have employed DFT/B3LYP calculations to highlight the molecular structural characteristics along with the electronic and spectroscopic properties of the newly prepared isomers, **2a** and **2b**. Additionally, the bioactivities of the newly synthesized compounds against some fungi and bacteria were investigated in vitro.

## Results and discussion

### Chemistry

Shown in Scheme 1 are reactions involved in the synthesis of compounds **2**, **3**, and **4**. 5-Acetyl-4-methyl-2-phenylamino-thiophene-3-carboxylic acid ethyl ester (**2**), a synthone required in this work, was prepared and characterized according to a procedure outlined by Mabkhot et al. [13] that involved stirring a mixture of ethyl acetoacetate and anhydrous potassium carbonate followed by addition of phenyl isocyanate and then chloroacetone. Compound **2**, on the other hand, was prepared in 90% yield (75% **2a** and 15% **2b**) from the reaction of compound **1** with bromine in glacial acetic acid as a solvent. Condensation of 2-aminobenzimidazole and compound **2** in ethanol containing triethylamine under reflux afforded compound **3** [14], whereas treatment of compound **2** with 3-amino-1,2,4-triazol in ethanol under reflux for 7 h yielded compound **4**. Structures of compounds **2**, **3**, and **4** where confirmed with the aid of IR, ^1^H NMR and ^13^C NMR spectra and with mass spectrometry, where the NMR spectra were in total agreement with the assigned structures. Similarly, mass spectra displayed the molecular ions corresponding to the respective molecular formulas of prepared compounds.

When compound **2** was prepared, we noticed that part of it dissolves in ethanol. Therefore, when it was recrystallized from this solvent followed by slow evaporation of ethanol, compound **2b** was obtained as crystals. This compound was characterized by NMR and x-ray crystallography. In the ^1^H NMR spectrum, the signal at *δ* 3.47 ppm has disappeared and a new signal due to a methyl group appeared instead at *δ* 2.45 ppm. Moreover, the aromatic region in the new compound was different from that of **2a**. Compound **2a** was obtained via a typical bromination of α-hydrogen of the methyl group next to the carbonyl group. However, bromination was also possible on the activated benzene ring; due to steric effect, substitution took place at the *para* rather than the *ortho* position, leading to the formation of compound **2b** (formation of compound **2b** was achieved via an electrophilic aromatic substitution reaction).

### Crystal structure of compound *2*

**Fig. 2:**
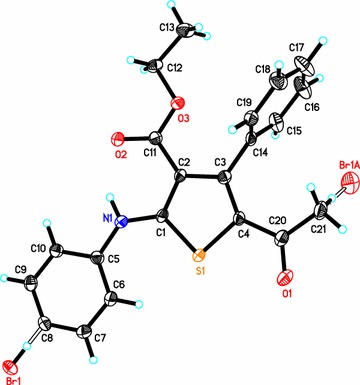
The ORTEP diagram of compound **2**. Displacement ellipsoids are *plotted* at the 50% probability level for non-H atoms showing the two different isomers

**Fig. 3:**
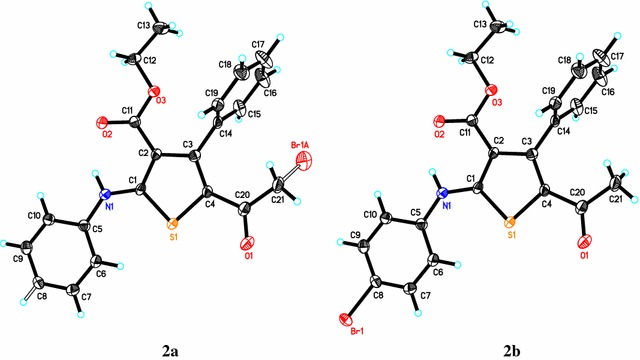
ORTEP diagram of the titled compound showing the two isomers, **2a** and **2b**, separately for clarification

**Fig. 4:**
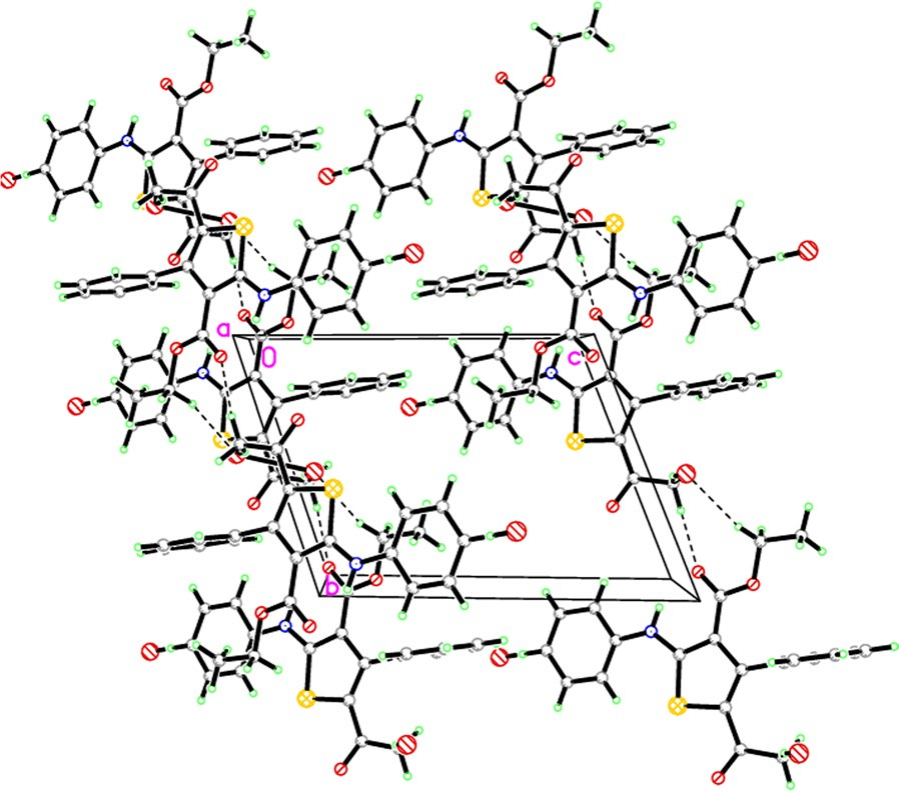
A view along the *b* axis of the crystal packing of compound **2**. *Dashed lines* indicate week hydrogen bonds

In the crystal structure of compound **2**, the asymmetric unit consists of one independent molecule with disorder in the position of bromine atom which eventually leads to two different isomers, **2a** (Br is on the side-chain) and **2b** (Br is on the benzene ring). Crystal structure of compound **2** is shown in Fig. 2, whereas depicted in Fig. 3 are the two isomers **2a** and **2b** for comparison. In the crystal structure of **2**, the phenyl ring (C14–C19) is nearly perpendicular to the central thiophene ring (C1–C4/S1) with a dihedral angle of 88.11°. On the other hand, the second phenyl ring (C5–C10) is coplanar with the central thiophene ring with a dihedral angle of 3.27°. All bond lengths and angles are in the normal range [15]. In addition, the two isomers contain strong intramolecular hydrogen bonds between H1N1 and O2 1.934 (9) and 2.650 (12) Å for N–H–O and N–O, respectively, Fig. 4. Crystallographic data and refinement information for compound **2** are summarized in Table 1.

**Table 1:** Crystal data and structure refinement for **2**

Chemical formula	C_21_H_18_BrNO_3_S
M_r_	444.25
Crystal system, space group	Triclinic, *P*-1
Temperature (K)	100
*a*, *b*, *c* (Å)	8.8152 (8), 10.0958 (9), 12.6892 (10)
α, β, γ (°)	68.549 (5), 81.667 (5), 68.229 (5)
V (Å3)	976.04 (15)
*Z*	2
Radiation type	Mo Kα
*µ* (mm^−1^)	2.23
Crystal size (mm)	0.20 × 0.15 × 0.07
Data collection	
Diffractometer	Bruker Kappa APEXII Duo diffractometer
Absorption correction	Numerical Blessing, 1995
T_min_, T_max_	0.717, 0.854
No. of measured, independent and observed [I > 2σ(I)] reflections	25,229, 3426, 2904
R_int_	0.055
Refinement	
R[*F* ^*2*^ > 2σ(*F* ^*2*^)], wR(*F* ^*2*^), S	0.046, 0.141, 1.06
No. of reflections	3426
No. of parameters	255
No. of restraints	0
H-atom treatment	H atoms treated by a mixture of independent and constrained refinement
Δρ_max_, Δρ_min_ (e Å^−3^)	1.3, −0.7

### Energetic and thermodynamic parameters

**Fig. 5:**
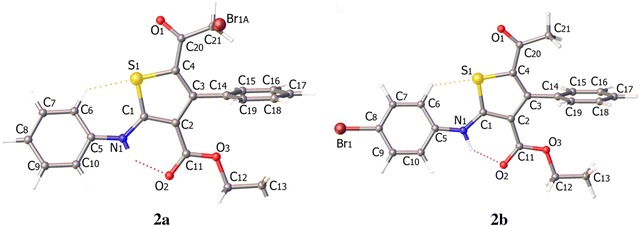
The optimized structures of studied compounds

The calculated total energy (E_tot_), zero point correction (ZPVE), and thermodynamic parameters such as enthalpy (H), entropy (S) and Gibbs free energy (G) for the two isomers **2a** and **2b** are listed in Table 2. The optimized structure of these isomers is given in Fig. 5. Both isomers are stabilized by intramolecular H-bonding interactions of the type N–H–O. To account for the extra stability of **2b** compared to **2a**, we employed the data presented in Table 1. Results of energy analysis show that **2b** has lower energy than **2a** by 3.51 kcal/mol, hence, **2b** represents the stable isomer of compound **2**. Using the equation K = e^−(∆G/RT)^, where the gas constant (R) is 2 × 10^−3^ kcal/mol k, the temperature (T) is 298.15 k, and the quantity ∆G is the difference between the Gibbs free energies of **2a** isomer relative to **2b**, we calculated the mole fractions of the two isomers to be 99.6 and 0.4 for **2b** and **2a**, respectively. These values confirm the predominance of **2b**.

**Table 2:** The calculated energies and thermodynamic parameters of the studied isomers of **2**

Parameter	**2a**	**2b**
E (a.u.)	−4063.8089	−4063.8145
ZPVE (a. u.)	0.3423	0.3437
S (cal mol^−1^ K^−1^)	182.2	182.5
∆G (kcal/mol)	−3.2919	0.0000
*μ* (Debye)	4.95	5.95
% POP	0.4	99.6

The calculated optimized structural parameters of the studied isomers are given in Table 3. Both calculated structures differ geometrically in the plane–plane dihedral angels, affording the three planes C14–C15–C16–C17–C18–C19, S1–C1–C2–C3–C4, and C5–C6–C7–C8–C9–C10. Both disorders (**2a** and **2b**) have the same dihedral angles but differ in the X-ray structure. This can be explained by two factors: 1) the crystallographic structure is an averaged structure 2) Gas phase calculations omit the packing interactions, therefore we are comparing solid state with gas phase which has more degrees of freedom. Another feature is the intramolecular hydrogen bonding, both disorders are stabilized by these H-bonding interaction of the type N–H–O (calculated 1.798 and 1.796 Å; experimental 1.934 Å) and by non-classical interaction C–H–S (calculated 2.487 and 2.479; experimental 2.480).

**Table 3:** The geometric parameters of both disorders, **2a** and **2b** (calculated and experimental)

	DFT		Exp.		DFT		Exp.
	**2a**	**2b**			**2a**	**2b**	
C21Br1(a)	–	1.917	1.897	C15–C14–C19	119.2	119.1	119.9
C8–Br1(b)	1.983	–	1.573	C15–C16–C17	120.3	120.2	120.0
O2–C11	1.228	1.228	1.225	C16–C17–C18	119.7	119.7	120.4
O3–C11	1.338	1.340	1.326	C17–C18–C19	120.1	120.2	119.9
O3–C12	1.452	1.451	1.460	C1–C2–C11	119.7	119.8	120.1
O1–C20	1.224	1.224	1.231	C1–C2–C3	112.3	112.2	112.7
N1–C1	1.350	1.354	1.360	C1–N1–C5	132.5	132.6	132.5
N1–C5	1.405	1.401	1.404	C1–S1–C4	91.4	91.3	91.2
S1–C1	1.736	1.736	1.726	C2–C11–O3	114.8	114.7	114.4
S1–C4	1.769	1.768	1.749	C2–C3–C14	123.9	124.4	123.9
C1–C2	1.419	1.416	1.391	C2–C3–C4	112.8	112.9	111.8
C2–C3	1.433	1.437	1.434	C3–C14–C15	120.9	120.4	121.3
C3–C4	1.382	1.378	1.373	C3–C14–C19	119.8	120.5	118.9
C2–C11	1.467	1.465	1.467	C3–C2–C11	128.0	128.0	126.6
C3–C14	1.493	1.493	1.486	C3–C4–C20	135.6	135.2	133.8
C4–C20	1.462	1.472	1.479	C4–C20–C21	121.7	121.3	120.4
C5–C6	1.401	1.401	1.393	C4–C3–C14	123.2	122.8	124.2
C5–C10	1.404	1.405	1.410	C5–C10–C9	120.6	121.1	120.7
C6–C7	1.393	1.392	1.382	C5–C6–C7	119.8	120.4	120.4
C7–C8	1.391	1.388	1.377	C6–C5–C10	119.0	118.6	118.6
C8–C9	1.395	1.393	1.392	C6–C7–C8	121.1	120.1	120.3
C9–C10	1.388	1.387	1.372	C7–C8–C9	119.1	120.5	120.4
C12–C13	1.514	1.514	1.502	C8–C9–C10	120.4	119.4	119.7
C14–C19	1.400	1.398	1.396	N1–C1–C2	123.7	123.5	123.2
C14–C15	1.397	1.398	1.384	N1–C5–C10	116.3	116.5	115.5
C15–C16	1.393	1.393	1.392	N1–C5–C6	124.7	124.9	125.9
C16–C17	1.393	1.394	1.374	O1–C20–C21	118.5	120.3	121.1
C17–C18	1.394	1.394	1.392	O1–C20–C4	119.8	118.4	118.6
C18–C19	1.393	1.392	1.386	O2–C11–C2	123.6	123.7	123.0
C20–C21	1.521	1.514	1.501	O2–C11–O3	121.6	121.6	122.6
N1–H–O2	1.798	1.796	1.934	O3–C12–C13	107.4	107.4	106.2
C6–H–S1	2.487	2.479	2.480	S1–C1–C2	111.7	111.7	111.9
Br1a–C21C20		–	126.3	S1–C1–N1	124.7	124.8	124.9
Br1–C8–C7	–	119.8	119.8	S1–C4–C20	112.5	112.9	113.7
Br1–C8–C9	–	119.7	119.9	S1–C4–C3	111.8	111.9	112.4
C11–O3–C12	116.5	116.6	116.6	θ_p1p2_	70.0	73.6	89.5
C14–C15–C16	120.3	120.5	119.6	θ_p1p3_	89.1	90.5	88.1
C14–C19–C18	120.4	120.5	119.8	θ_p2p3_	19.1	16.9	3.3

*θ* the dihedral angle between two planes, *p1* C14–C15–C16–C17–C18–C19, *p2* S1–C1–C2–C3–C4, *p3* C5–C6–C7–C8–C9–C10

#### Antibacterial and antifungal activity

We investigated the in vitro antibacterial and antifungal activity of the newly synthesized compounds against two Gram-positive (*Streptococcus pneumoniae* and *Bacillis subtilis*) and two Gram-negative bacteria (*Pseudomonas aeruginosa* and *Escherichia coli*) which are known to cause infections in humans. On the other hand, the antifungal activity of these compounds was assessed against four fungal species; *Aspergillus fumigates*, *Syncephalastrum racemosum*, *Geotricum candidum*, and *Candida albicans*. Activity against those pathogens was expressed as diameter of the inhibition zone, in mm, using the well-diffusion agar method. In this investigation, we have employed ampicillin, gentamicin, and amphotericin B as standard antimicrobial agents to compare the potency of the tested compounds. Results from this study are shown in Table 4.

**Table 4:** Antibacterial and antifungal activity of compounds **2**, **3**, and **4** (diameter of inhibition zone is given in mm)

A) Antifungal activity				
Tested pathogen	FUNGI			
	*A. fumigates*	*S. racemosum*	*G. candidum*	*Candida albicans*
	Amphotericin B			
Reference compound	23.7 ± 0.1	19.7 ± 0.2	28.7 ± 0.2	25.4 ± 0.1
**2**	16.2 ± 0.4	15.0 ± 0.4	17.6 ± 0.6	NA
**3**	21.3 ± 0.4	17.2 ± 0.2	24.6 ± 0.6	NA
**4**	17.6 ± 0.6	15.4 ± 0.3	12.6 ± 0.4	NA

B) Antibacterial activity				
Tested pathogen	Gram positive bacteria		Gram negative bacteria	
	*S. pneumoniae*	*B. subtilis*	*P. aeruginosa*	*E. coli*
	Ampicillin		Gentamicin	
Reference compounds	23.8 ± 0.2	32.4 ± 0.3	17.3 ± 0.1	19.9 ± 0.3
**2**	16.9 ± 0.6	18.2 ± 0.4	NA	11.9 ± 0.6
**3**	18.2 ± 0.1	20.3 ± 0.1	NA	20.3 ± 0.1
**4**	12.3 ± 0.6	12.7 ± 0.4	NA	8.5 ± 0.4

Results in Table 4 reveal that compound **3** has remarkable activity against the tested fungi *A. fumigates*, *S. racemosum*, and *G. candidum*, whereas compounds **2** and **4** exhibited moderate activities against these fungi. On the other hand, compound **3** displayed significant activity against the gram positive bacterial strains *S. pneumoniae* and *B. subtilis* and showed excellent activity against the gram negative strain *E. coli*. Compounds **2** and **4** showed moderate activities against the aforementioned bacterial strains. In addition, results suggest that the new skeletons possessing benzimidazole and thiophene moieties may provide valuable leads for the synthesis and development of novel antimicrobial agents. Moreover, compound **3** could be a promising antifungal and antibacterial agent, however, more work is needed to evaluate the safety and efficacy of this compound.

## Experimental

### Reagents and instrumentation

Reagents used throughout this work were obtained from commercial sources and were used as received without further purification. Progress of reactions was monitored with TLC using Merck Silica Gel 60 F–254 thin layer plates (Billerica, MA, USA). Infrared Spectra were recorded, as KBr pellets, on a Nicolet 6700 FT-IR Nicolet spectrophotometer (Madison, WI, USA). Melting points were determined on a Gallenkamp apparatus in open glass capillaries and are uncorrected. We acquired ^1^H- and ^13^C-NMR spectra with a Varian Mercury Jeol-400 NMR spectrometer (Akishima, Japan) with CDCl_3_ as solvent. Chemical shifts are reported in ppm (δ) relative to tetramethylsilane as an internal reference and coupling constants, J, are given in Hz. Mass spectral data were obtained with the aid of a Jeol of JMS-600H mass spectrometer (Tokyo, Japan). Single-crystal X-ray diffraction measurements were performed using a Bruker SMART APEX II CCD diffractometer (Karlsruhe, Germany). Elemental analyses were performed on a Euro Vector Elemental Analyzer (EA 3000 A, Via Tortona, Milan, Italy).

### Synthesis of 5-(2-bromo-acetyl)-4-phenyl-2-phenylamino-thiophene-3-carboxylic acid ethyl ester (2)

Compound **2a** was synthesized according to the following general procedure: A solution of 5-acetyl-4-phenyl-2-phenylaminothiophene-3-carboxylic acid ester (**1**) (3.0 g, 10 mmol) in glacial acetic acid (100 mL) was heated to 90–100 °C with vigorous stirring. To this hot solution, bromine (1.1 ml) in glacial acetic acid (50 mL) was added dropwise over a period of 30 min. After complete addition of bromine, the reaction mixture was stirred vigorously at room temperature for further 2 h until evolution of hydrogen bromide gas ceased, then was poured onto ice. The solid product was collected by filtration, washed with water, dried, and recrystallized from ethanol to give **2** as white yellowish crystals. Yield 75%; m.p.: 120–122 °C; IR (KBr): 3452 (NH), 1655 (C=O), 1633 (C=O) cm^−1^. ^1^H NMR (400 MHz, CDCl_3_): δ 0.72 (t, *J* = 6.0 Hz, 3H, CH_3_–CH_2_), 3.47 (s, 2H, CH_2_–Br), 3.91 (q, *J* = 6.1 Hz, 2H, CH_2_–CH_3_), 7.21–7.51 (m, 10H, aromatic), 10.81 (s, 1H, NH–ph). ^13^C NMR (100 Hz, CDCl_3_): δ 28.7 (CH_3_), 33.0 (CH_2_Br), 60.1 (CH_2_O), 110.5, 117.8, 120.5, 121.8, 125.2, 128.3, 129.8, 132.7, 136.7, 138.3. 139.2, 147.8, 166.3 (C=O), 184.4 (C=O). Anal. calcd. For C_21_H_18_BrNO_3_S: C, 56.76; H, 4.08; N, 3.15; S, 7.22; Found: C, 56.66; H, 3.98; N, 3.18; S, 7.34.

Compound **2b**. Yield 15%; ^1^H NMR (400 MHz, DMSO-d_6_): *δ* 0.88 (t, *J* = 6.0 Hz 3H, CH_3_–CH_2_), 2.45 (s, 3H, CH_3_), 3.98 (q, *J* = 6.2 Hz, 2H, CH_2_–CH_3_), 7.45-7.83 (m, 9H, aromatic), 10.48 (s, 1H, NH–amine), ppm. ^13^C NMR (100 Hz, DMSO-d_6_): *δ* 11.9 (CH_3_), 12.0 (CH_3_), 60.0 (CH_2_), 111.2, 113.2, 118.3, 119.2, 122.8, 123.0, 127.8, 132.3, 134.0, 137.8, 150.0, 165.2 (C=O), 180.0 (C=O).

### Synthesis of 4-phenyl-2-phenylamino-5-(1H-1,3-a,8-triaza-cyclopenta[α]inden-2-yl)-thiophene-3-carboxylic acid ethyl ester (3)

The following procedure was employed to prepare the title compound: A mixture of compound **2** (0.44 g, 1 mmol) and 2-aminobenzimidazole (0.133 g, 1 mmol) was refluxed in ethanol (15 mL) for 8 h in the presence of 0.5 mL of triethylamine (TEA). After cooling, the solid product was collected by filtration to afford the title compound **3** as a yellow powder. Yield 82%; m.p.: 146–148 °C; IR (KBr): 3452 (NH), 1633 (C=O), 1586 (C=N) cm^−1^. ^1^H NMR (400 MHz, CDCl_3_): *δ* 0.95 (t, *J* = 6.0 Hz 3H, CH_3_–CH_2_), 3.25 (q, *J* = 6.1 Hz, 2H, CH_2_–CH_3_), 6.57–7.51 (m, 14 H, aromatic), 7.54 (s, 1H, CH-imidazo), 10.73 (s, 1H, NH–ph) 10.81 (s, 1H, NH) ppm. ^13^C NMR (100 Hz, CDCl_3_): *δ* 12.1 (CH_3_), 54.5 (CH_2_), 111.0, 119.4, 119.7, 120.0, 126.2, 127.3, 128.0, 131.0, 135.0, 153.0, 164.9 (C=O). MS *m/z* 478 [M^+^, 1.2%] calcd. for C_28_H_22_N_4_O_2_S; 442 (18.9%); 328 (22.6%), 112 (100%); Anal. calcd. For C_28_H_22_N_4_O_2_S: C, 70.27; H, 4.63; N, 11.71; S, 6.70; Found: C, 70.50; H, 4.53; N, 11.66; S, 6.84.

### Synthesis of 5-(1H-Imidazo[1,2-b][1,2,4]triazol-5-yl)-4-phenyl-2-phenylamino-thiophene-3-carboxylic acid ethyl ester (4)

Compound **4** was prepared according to the procedure employed for the synthesis of compound **3** with some modifications: a mixture of compound **2** (0.44 g, 1 mmol) and 3-amino-1*H*-1,2,4-triazole (0.84 g, 1 mmol) was heated under reflux for 8 h in ethanol (10 mL) in the presence of 0.5 mL of trimethylamine (TEA). The solid product was collected by filtration to afford the desired product as a brown powder. Yield 49%; mp 150–152 °C; IR (KBr): 3409 (NH), 1658 (C=O), 1627 (C=N), 1586 cm^−1^ (C=C). ^1^H NMR (400 MHz, CDCl_3_): *δ* 0.69 (t, *J* = 6.0 Hz 3H, CH_3_–CH_2_), 3.52 (q, *J* = 6.0 Hz, 2H, CH_2_–CH_3_), 5.14 (s, 1H, NH–amine), 7.24–7.53 (m, 14 H, aromatic), 7.56 (s, 1H, CH–imidazol), 10.74 (s, 1H, CH–triazol) 10.85 (s, 1H, NH–triazol) ppm. ^13^C NMR (100 Hz, CDCl_3_): *δ* 12.1 (CH_3_), 54.8 (CH_2_), 119.1, 119.9, 120.0, 121.3, 125.0, 126.9, 127.2, 127.3, 127.5, 128.1, 128.7, 128.9, 131.6, 131.9, 148.5, 148.7, 164.8 (C=O). MS *m/z* 429 [M^+^, 81.3%] calcd. for C_23_H_19_N_5_O_2_S; 275 (53.8%); 211 (47.4%); 91 (100%); Anal. calcd. For C_23_H_19_N_5_O_2_S: C, 64.32; H, 4.46; N, 16.31; S, 7.47; Found: C, 64.55; H, 4.39; N, 16.50; S, 7.66.

### X-ray measurements

Crystals of compound of **2** were obtained by slow evaporation from an ethanol solution at room temperature. Crystallographic data were collected on a Bruker Kappa APEXII Duo diffractometer, equipped with graphite monochromatic Mo *K*α radiation, λ = 0.71073 Å at 100 (2) K. Cell refinement and data reduction were accomplished with the aid of a Bruker SAINT, whereas structure was solved by means of SHELXT [16, 17]. The final refinement was carried out by full-matrix least-squares techniques with anisotropic thermal data for nonhydrogen atoms on *F*2. CCDC 1450887 contains the supplementary crystallographic data for compound **2** and can be obtained free of charge from the Cambridge Crystallographic Data Centre via http://www.ccdc.cam.ac.uk/data_request/cif.

### Computational details

X-ray structure coordinates of the two isomers of **2** were employed as input files for comparing their relative stability. Structure optimizations were accomplished using the B3LYP method and 6‒311G(d,p) basis set with the aid of Gaussian 03 software [18]. The optimized geometries gave no imaginary vibrational modes. GaussView4.1 [19] and Chemcraft [20] programs have been employed to extract the calculation results and to visualize the optimized structures.

### Antimicrobial activity

In vitro antibacterial screening tests of the newly synthesized compounds were performed against four bacterial strains: two Gram-positive (*Streptococcus pneumonia* and *Bacillis subtilis*) and two Gram-negative (*P. aeruginosa* and *E. coli*) in addition to four different fungi; *A. fumigates*, *S. racemosum*, *G. candidum*, and *C. albicans*. The disc diffusion method [21] was used in this assay and each experiment was performed in triplicate; experimental details of these techniques can be found elsewhere [22, 23]. Readings of the zone of inhibition, which are shown in Table 4, represent the mean value of three readings. Amphotericin B, ampicillin, and gentamicin were employed as standard drugs in this assay.

## Conclusions

Three new thiophene derivatives were synthesized in good yield. These newly synthesized compounds were characterized by means of different spectroscopic methods and by elemental analysis. Furthermore, X-ray crystallography was performed on the two isomeric forms of compound **2** in addition to DFT and energy calculations to show the dominance of one of the isomers over the other. Additionally, the new compounds were screened for their antimicrobial activity against a number of bacterial and fungal strains. Results showed that compound **3** was a promising candidate as a potential antibacterial and antifungal agent; it exhibited remarkable activity against the studied bacterial strains, especially the gram negative bacteria *E. coli* in addition to some fungi. More work is needed to evaluate its safety and efficacy.

## References

[1] Dimmock JR, Puthucode RN, Smith JM, Hetherington M, Quail JW, Pugazhenthi U, Lechler T, Stables J (1996). (Aroyloxy)aryl semicarbazones and related compounds: a novel class of anticonvulsant agents possessing high activity in the maximal electroshock screen. J Med Chem.

[2] Ragavendran J, Sriram D, Patil S, Reddy IV, Bharathwajan N, Stables J, Yogeeswari P (2007). Design and synthesis of anticonvulsants from a combined phthalimide-GABA-anilide and hydrazone pharmacophore. Eur J Med Chem.

[3] Polivka Z, Holubek J, Svatek E, Metys J, Protiva M (1984). Potential hypnotics and anxiolytics: synthesis of 2-bromo-4-(2-chlorophenyl)-9-/4-(2-methoxyethyl)-piperazino0-6H-thieno/3,2-f/-1,2,4-triazolo/4,3-a/-1,4-diazepine and of some related compounds. Collect Czech Chem Commun.

[4] Yogeeswari P, Thirumurugan R, Kavya R, Samuel JS, Stables J, Siram D (2004). 3-Chloro-2-methylphenyl-substituted semicarbazones: synthesis and anticonvulsant activity. Eur J Med Chem.

[5] Gunizkuculguzel S, Mazi A, Sahin F, Qzturk S, Stables J (2003). Synthesis and biological activities of diflunisal hydrazide–hydrazones. Eur J Med Chem.

[6] Thirumurugan R, Sriram D, Saxena A, Stables J, Yogeeswari P (2006). 2,4-Dimethoxyphenylsemicarbazones with anticonvulsant activity against three animal models of seizures: synthesis and pharmacological evaluation. Bioorg Med Chem.

[7] Riaz N, Anis I, Rehman A, Malik A, Ahmed Z, Muhammad P, Shujaat SA, Ur-Rahman A (2003). Emodinol, β-Glucuronidase, inhibiting triterpine from *Paeonia emodi*. Nat Pro Res.

[8] Shank RP, Doose DR, Streeter AJ, Bialer M (2005). Plasma and whole blood pharmacokinetics of topiramate: the role of carbonic anhydrase. Epilepsy Res.

[9] Ahmad VU, Khan A, Farooq U, Kousar F, Khan SS, Nawaz SA, Abbasi MA, Choudhary MI (2005). Three New cholinesterase-inhibiting *cis*-clerodane diterpenoids from *Otostegia limbata*. Chem Pharm Bull.

[10] Ahmad VU, Abbasi MA, Hussain H, Akhtar MN, Farooq U, Fatima N, Choudhary MI (2003). Phenolic glycosides from *Symplocos racemosa*: natural inhibitors of phosphodiesterase I. Phytochemistry.

[11] Mabkhot YN, Aldawsari FD, Al-Showiman SS, Barakat A, Soliman SM, Choudhary MI, Yousuf S, Mubarak MS, Ben Hadda T (2015). Novel enaminone derived from thieno[2,3-b]thiene: synthesis, x-ray crystal structure, HOMO, LUMO, NBO analyses and biological activity. Chem Cent J.

[12] Mabkhot YN, Aldawsari FD, Al-Showiman SS, Barakat A, Ben Hadda T, Mubarak MS, Sehrish N, Ul-Haq Z, Rauf A (2015). Synthesis, bioactivity, molecular docking and pom analyses of novel substituted thieno[2,3-*b*]thiophenes and related congeners. Molecules.

[13] Mabkhot YN, Kaal NA, Alterary S, Al-Showiman SA, Barakat A, Ghabbour HA, Frey W (2015). Synthesis, in vitro antibacterial, antifungal, and molecular modeling of potent anti-microbial agents with a combined pyrazole and thiophene pharmacophore. Molecules.

[14] Takagi H, Kobayashi S, Kamioka T, Kamoshita K (1972). Studies on heterocyclic compounds. 10^1^. Synthesis of some imidazo[1,2-a]benzimidazoles with potent analgetic activities. J Med Chem.

[15] Allen FH, Kennard O, Watson DG, Brammer L, Orpen AG, Taylor R (1987). Tables of bond lengths determined by X-ray and neutron diffraction Part 1. J Chem Soc Perkins Trans.

[16] Sheldrick GM (2015). SHELXT-Integrated space-group and crystal-structure determination. Acta Cryst Sect A Found Adv.

[17] Brucker (2009). APEX2, SAINT and SADABS.

[18] Gaussian-03 (2004) Revision C.01. Gaussian Inc, Wallingford

[19] Gauss View (2007) Version 4.1. Semichem Inc, Shawnee Mission

[20] Chemcraft. Lite Version Build 08. http://www.chemcraftprog.com/. Accessed 1 Apr 2005

[21] Mabkhot YN, Alatibi F, El-Sayed NNE, Al-Showiman S, Kheder NA, Wadood A, Rauf A, Bawazeer S, Ben Hadda T (2016). Antimicrobial activity of some novel Armed thiophene derivatives and Petra/Osiris/Molinspiration (POM) analyses. Molecules.

[22] Jafri L, Ansari FL, Jamil M, Kalsoom S, Qureishi S, Mirza B (2012). Microwave-assisted synthesis and bioevaluation of some semicarbazones. Chem Biol Drug Des.

[23] Rehman A, Choudhary MI, Thomsen WJ (2001). Bioassay techniques for drug development.

